# Objective and automatic classification of Parkinson disease with Leap Motion controller

**DOI:** 10.1186/s12938-018-0600-7

**Published:** 2018-11-12

**Authors:** A. H. Butt, E. Rovini, C. Dolciotti, G. De Petris, P. Bongioanni, M. C. Carboncini, F. Cavallo

**Affiliations:** 1 BioRobotics Institute, Scuola Superiore Sant’Anna, Pontedera, Italy; 2 Institute of Information Science and Technologies National Research Council, Pisa, Italy; 3 Telecom Italia, WHITE Lab (Wellbeing and Health Innovative Technologies Lab), Pisa, Italy; 4 Severe Acquired Brain Injuries Department Section, Azienda Ospedaliera Universitaria Pisana, Pisa, Italy; 5 Neurocare Onlus, Pisa, Italy

**Keywords:** Objective diagnosis in Parkinson, Leap Motion, Motion analysis, Supervised learning, Features selection

## Abstract

**Background:**

The main objective of this paper is to develop and test the ability of the Leap Motion controller (LMC) to assess the motor dysfunction in patients with Parkinson disease (PwPD) based on the MDS-UPDRSIII exercises. Four exercises (thumb forefinger tapping, hand opening/closing, pronation/supination, postural tremor) were used to evaluate the characteristics described in MDS-UPDRSIII. Clinical ratings according to the MDS/UPDRS-section III items were used as target. For that purpose, 16 participants with PD and 12 healthy people were recruited in Ospedale Cisanello, Pisa, Italy. The participants performed standardized hand movements with camera-based marker. Time and frequency domain features related to velocity, angle, amplitude, and frequency were derived from the LMC data.

**Results:**

Different machine learning techniques were used to classify the PD and healthy subjects by comparing the subjective scale given by neurologists against the predicted diagnosis from the machine learning classifiers. Feature selection methods were used to choose the most significant features. Logistic regression (LR), naive Bayes (NB), and support vector machine (SVM) were trained with tenfold cross validation with selected features. The maximum obtained classification accuracy with LR was 70.37%; the average area under the ROC curve (AUC) was 0.831. The obtained classification accuracy with NB was 81.4%, with AUC of 0.811. The obtained classification accuracy with SVM was 74.07%, with AUC of 0.675.

**Conclusions:**

Results revealed that the system did not return clinically meaningful data for measuring postural tremor in PwPD. In addition, it showed limited potential to measure the forearm pronation/supination. In contrast, for finger tapping and hand opening/closing, the derived parameters showed statistical and clinical significance. Future studies should continue to validate the LMC as updated versions of the software are developed. The obtained results support the fact that most of the set of selected features contributed significantly to classify the PwPD and healthy subjects.

## Background

Parkinson disease (PD) is a degenerative disorder of the central nervous system and is currently growing as a neurological disorder in the aging society. According to the Parkinson Disease Foundation, one million Americans are living with Parkinson disease, and approximately 60,000 Americans are diagnosed with Parkinson disease each year. Similarly, 1.2 million Europeans suffer from it, and this number forecasted to double by 2030 [1].

Parkinson disease symptoms can be classified as motor or non-motor. The first includes tremor, rigidity, bradykinesia, and postural instability. Non-motor symptoms are various and includes loss of taste and sense of smell, sleep disturbances, gastrointestinal complications, constipation, swallowing problems, anxiety, pain, fatigue, depression, sexual dysfunction, hallucinations and psychosis, impulse control disorders, cognitive impairment, and dementia [2].

Currently, motor disorder studies still offer different clinical challenges for the scientific community. For example, diagnostic criteria for PD rely on the presence of motor signs and, for the assessment of movement disorders, the neurologist uses a visual examination of motor tasks and semi-quantitative rating scales, such as the Hoehn–Yahr (HY) Scale and the Movement Disorder Society-Unified Parkinson’s Disease Rating Scale (MDS-UPDRS). This subjective assessment leads to inter- and intra-rater variability due to neurologist subjectivity; therefore, the necessity of objective motor assessment tools is crucial in the future of PD diagnostic procedures [3].

Similarly, motor performance and pattern analysis and interpretation seem to have relevance in early diagnosis [4]. Patients generally are clearly diagnosed with PD at the advanced stage; moreover, any neuroprotective therapy initiated at such a late stage may have fewer substantial effects on the disease progression. Thus, the investigation of movement performance is one of the most challenging opportunities to obtain valid and objective biomarkers to recognize early PD symptoms [5].

Additionally, motor assessment is valuable in differential diagnosis between PD and atypical parkinsonism and can be more challenging particularly at early stages, when clinical features can overlap and in cases of misdiagnosis, as described in several clinicopathological studies [6]. Moreover, factors including the long-term development of the disease, often characterized by motor fluctuations during specific times of day; long waiting lists; and high travel costs (particularly for people who live in rural areas) support the need for specific monitoring instrumentation to monitor PD progression also at home [7].

Currently, no cure is available for the disease or the symptoms; therefore, movement therapy is important to delay the loss of motor function. However, the most developed solutions in providing an objective assessment involve neuroimaging. Many imaging methods have been employed for the diagnosis of PD, the most common being positron emission tomography (PET) and single-photon emission computed tomography (SPECT) [8]. However, due to their high level of invasiveness and prohibitive cost, less expensive alternative techniques are required. Recent advances in technology have enabled human motion analysis based on sensor data, which can be used to extract valuable information for assessing movement disorders in lower and upper limbs. While a number of works have focused on solutions for lower limbs, few studies are present in literature that aim to deeply investigate solutions for motor assessment in upper limbs [3]. For this reason, a solution for upper limbs is provided here.

In the last decade, several motion sensing devices have emerged, including wearable sensors (e.g., accelerometer, gyroscope) and vision-based sensors (e.g., Vicon, Microsoft Kinect) [9]. Wearable sensors, such as inertial measurement units (IMUs), offer a more portable, flexible, inexpensive solution for the assessment of motor dysfunction of PD. The advantages of accelerometers include small size and relatively high sampling frequency. Their main disadvantage is that they need to be attached to the body, which may affect their motion performance. Indeed, for each gram of additional mass a sensor adds, the peak frequency of finger tremor decreases up to 0.85 Hz, which affects the acceleration amplitude [10].

Recently, several studies [11–13] were conducted on PwPD to assess motor dysfunction using commercially available non-contact video and RGB-D based sensors. Result showed that the video-based system could detect bradykinesia and dyskinesia in the clinical environment. Recent studies also focused on another commercially available device, the Leap Motion controller (LMC), which incorporates a 3D camera that is connected to a computer and is claimed to measure positional data accurate to within 0.01 mm [14]. LMC was primarily employed with video games, such as in the study of Lin [13], where it was used to track the hand movements to enhance the computer accessibility in rehabilitation. Similarly, in the study of Blazica [15], LMC was used with kinetic sensor to achieved the full kinematics of movement during game play. LMC has also been used for the assessment of movement disorder in PD. However, to the best of our knowledge, after a literature review, the number of studies focusing on the assessment of motor dysfunction in PD with LMC are limited. A study by Matthew [10] was performed using Leap Motion and a tremor-scope accelerometer-based device to assess the rest tremor and essential tremor in PwPD and healthy control, comparing K-mean clustering and SVM techniques. Results showed that out of eight, six characteristics of frequency and power showed no statistical difference between devices according to the Wilcoxon Signed Ranks Test and Sign Test.

Similarly, in another study by Nastaran [16], a kinetic sensor and LMC were tested on real patients to understand the ability of both systems. For motor examination of Parkinson’s patients by considering the limitations of the Kinect sensor, some part of the motor section was chosen to be studied by the Kinect sensor, such as leg agility, arising from chair, postural stability, and body bradykinesia and hypokinesia. Since the used Kinect sensor is not able to give precise information of the hand and finger movements, the Leap Motion controller was implemented to compensate for this specific limitation of the Kinect sensor. From Leap Motion, different characteristics of the hand movements were extracted. The hand movements of the patients were based on the line created by moving the hand in the air. By comparing the line on the screen and the results from the patient exercise, the physician can see how shaky the hand is and study the rate of the tremors. Similarly, in another study by Kai-Hsiang Chena [17], the Leap Motion controller system was tested with four patients diagnosed with essential tremor. The results showed that the system has the potential to assess the hand tremor in the clinical environment. This study did not include the other types of action tremors due to hardware limitation, and all examination were of Parkinson’s patients.

To the best of our knowledge, no comprehensive study, in which multiple exercises of section 3 of UPDRSIII are used on real PwPD with LMC, has been performed. This study encompasses the four exercises (hand opening/closing, thumb forefinger tapping, pronation/supination, and postural tremor) of section 3 of UPDRSIII motor tasks for the objective assessment of PwPD with LMC. To test the potential of the LMC, we first extracted the characteristics of UPDRSIII from all the exercises objectively. Feature selection algorithms were employed to select the most discriminating features. These selected features were fused and entered in the machine learning algorithms to classify the PwPD and healthy control. In this context, the aim of this paper is to present a deeper investigation, with respect to the state of art, of the use of the LMC in clinical setting to assess motor performance in upper limbs; provide a comprehensive study of all the exercises of the MDS-UPDRS scale, including upper limbs; present algorithms to extrapolate movement parameters of each exercise that are, as much as possible, comparable with the visual assessment provided by the neurologist; provide a statistical investigation of the significance of the extracted features, focusing on their correlation with the clinical scores and their relevance with respect to a control group of healthy subjects; propose a machine learning model for classifying patients and healthy subjects on the basis of different feature selection methods; give indications about the opportunities and limitations of the LMC in PD applications, and profiles possible future investigations to improve the current study.

## Methods

### Participants

All subjects were recruited at the Cisanello Hospital, Pisa. Sixteen PwPD (11 men, 5 women; mean age ± SD: 68.8 ± 9.43 years old; average MDS/UPDRS-Section III scores ± SD: 19.69 ± 8.91; average Hoehn and Yahr (H &Y) stage ± SD: (1.6 ± 0.5) and 12 healthy controls (2 men, 10 women; mean ± SD: 70.2 ± 11.88 years old) were asked to participate in this study. Exclusion criteria were impairments or diseases other than PD (i.e. orthopaedic or neurologic) that could affect the task performance. All subjects lived independently in the community; the most relevant clinical information is described in Table 1. Upon arrival at the testing location, all subjects were informed of the purpose of the study and provided written consent.

**Table 1:** Clinical details of healthy control and patient with Parkinson disease

Healthy subjects	Patient with Parkinson disease			
Age (gender)	Age (gender)	UPDRS III (0–56)	H &Y (1–5)	Disease duration (years)
74 (M)	72 (M)	9	1	6
43 (F)	76 (M)	5	1	7
75 (F)	62 (M)	23	1.5	7
80 (M)	62 (M)	23	2	10
58 (F)	68 (F)	26	2	14
61 (F)	65 (F)	9	1.5	4
83 (F)	69 (M)	25	2.5	10
77 (F)	82 (M)	32	2.5	7
79 (F)	61 (F)	18	1.5	3
61 (F)	60 (F)	26	2	20
78 (F)	76 (M)	20	2	10
74 (F)	75 (M)	32	2	10
	82 (M)	15	1	1
	46 (F)	7	1	6
	63 (M)	15	1	2
	75 (M)	30	1.5	8
70.25 (± 11.88)	68.80 (± 9.43)	19.69 (± 8.91)	1.63 (± 0.53)	7.81 (± 4.71)

### Instruments

**Fig. 1:**
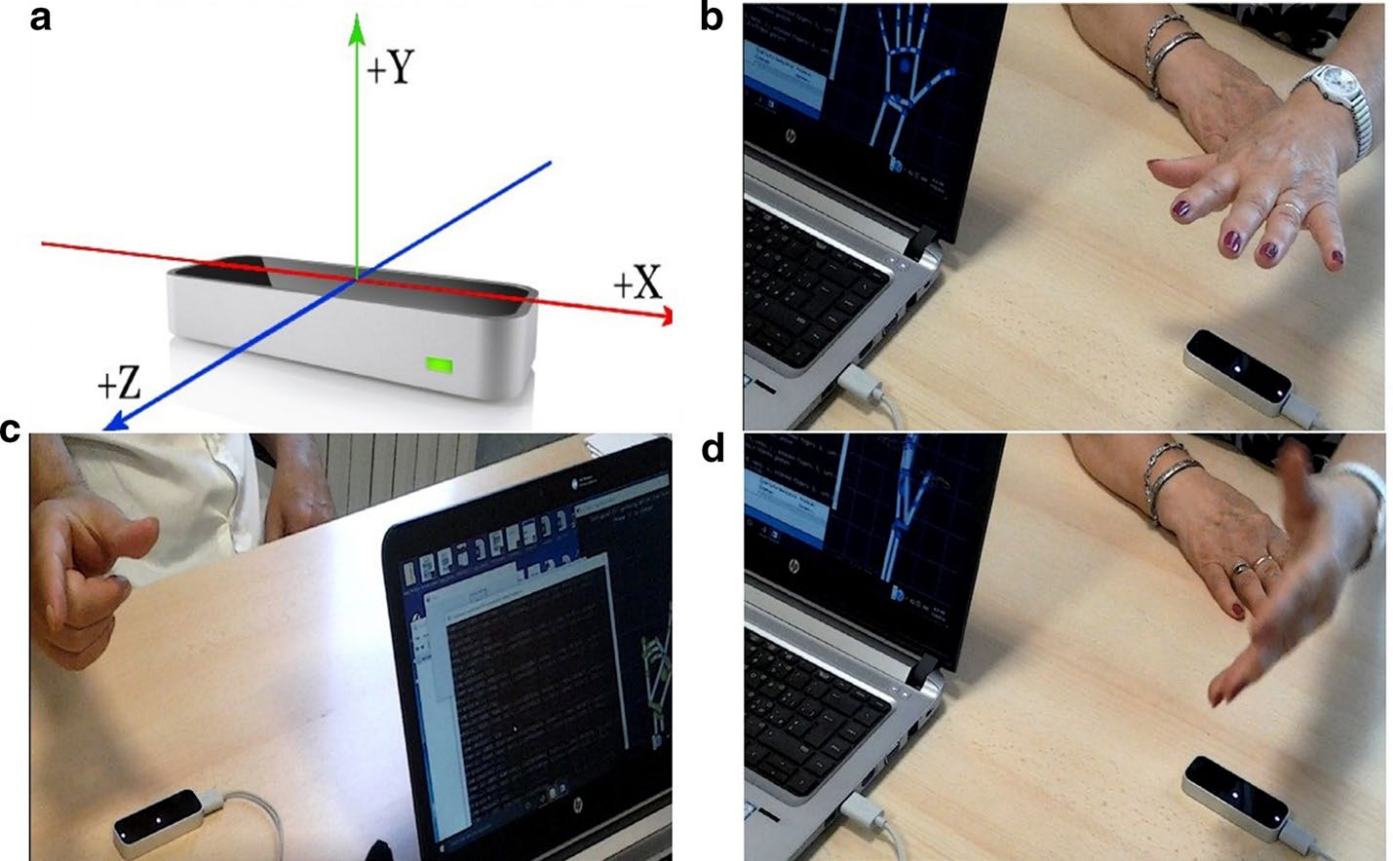
Coordinate system of LMC and experimental setup for each exercise: **a** leap motion controller,** b** postural tremor and hand opening–closing, **c** thumb fore-finger tapping, **d** forearm pronation/supination

LMC (Fig. 1) is a commercially available non-contact optical device manufacturer (LEAP MOTION, INC., USA), 45 g in weight, that can detect the motion and position of the hand in 3D. It consists of three infrared (IR) transmitters (LEDs) and two IR depth data capture cameras [16]. Both IR cameras are at a distance of 20 mm from the center of the LMC. The field of view in the hemispherical area is approximately 150°. The information regarding the user’s hand, fingers, and gestures is captured as long as the hand is between 25 and 600 mm above the center of the sensor. The carried hand position is relative to the center of the LMC. According to the manufacturer specifications, the accuracy of the LMC in spatial measurement can reach 0.01 mm; in previous studies, however, the error of spatial measurement in a static setup was approximately 0.2 mm, and the average spatial error in continuous motion was 0.4 mm [18]. The LMC was placed on the table (as shown in Fig. 1a) in all exercises. In this study, average sampling rate during the data acquisition was 35 Hz.

### Experimental protocol

The experimental protocol was composed of four exercises, corresponding to motor tasks 3.4, 3.5, 3.6, and 3.18 of the MDS/UPDRS-Section III [19]: pronation/supination of the forearms (PSUP); opening/closing of the hands (OPCL); thumb-forefinger tapping (THFF); and postural tremor (POST).

The subjects were asked to sequentially perform the exercises three times for both upper limbs to complete the experimental session. In addition, every subject underwent a short preliminary training to try all required movements. A neurologist assessed the subjects during the execution of the exercises and assigned them a score according to the tasks in MDS/UPDRS, based on the Edinburgh Handedness inventory [18] that is used to assess the dominance of a person’s right or left hand. Further dominant hand parameters were used for the analysis. These exercises were performed with both hands (left and right) for each exercise.

#### Pronation/supination (PSUP)

The subject was directed to assume a sitting posture at rest and was asked to put the arm outstretched in front of him or herself, with the wrist stable, the hand in prone position, and the fingers outstretched approximately 1 cm apart. After 5 s in resting position, the subject executed forearm pronation supination movements as fast and as wide as possible for 10 s. Then, the subject was directed to keep the hand in prone position and fingers outstretched in resting position for 5 s more.

#### Opening closing hand (OPCL)

The subject was directed to assume a sitting posture at rest with the arm flexed at the elbow. The elbow was fixed on the table, and the palm of the hand was kept in front of the subject. After 5 s in resting position, the subject alternatively opened and closed the hand as fast and with the fingers as wide as possible for 10 s, keeping the forearm and the wrist fixed. Then the subject was directed to keep the palm of the hand in front of the body in resting position for 5 s.

#### Thumb forefinger tapping (THFF)

The subject was directed to assume a sitting posture at rest, keeping the hand outstretched in front of him or herself. In the starting position, the thumb and the forefinger were in open position. The subject remained for 5 s in resting position and then tapped the forefinger against the thumb as quickly and as widely as possible for 10 s. Then, the subject was directed to keep the thumb and forefinger in open position for 5 s.

#### Postural tremor (POST)

The subject was directed to assume a sitting posture at rest and was asked to put the arm outstretched in front of him or herself with wrist stable, the hand in prone position, and the fingers outstretched approximately 1 cm apart from each other. The subject remained in this position for the duration of the exercise, for 20 s.

An alarm controlled by a timer on the PC was used to prompt the subjects to perform the correct movements in the different phases of the exercises.

### Data acquisition from Leap Motion

The LMC software development kit (SDK) (Fig. 1) device directly provides some of the most relevant points of hand movements, avoiding the need for complex computations to extract the depth and colour data [20]. A stand-alone C++ program was implemented to develop a simple user interface to record the movements and save data in CVS format for further signal processing. The code was run three times for each participant for both hands (right and left). Further, raw data was fed to the algorithms to extract the features. The LMC SDK was used to record the movements of the fingers and hand for each exercise, acquiring both the three-dimensional coordinates of fingertips and the following four features, computed directly from the algorithms included in the LMC SDK:

1. Palm angle: Roll angle of the palm, i.e. rotation of the hand around the z-axis.
  This value was used in the PSUP exercise and was computed via the LMC SDK instruction given below:
  1 $$A_{p} \left( k \right) = hand.palmNormal.roll\left( k \right)$$
  This angle is estimated as the angle between the negative y-axis and the projection of the palm normal vector into the x–y plane (Fig. 2c).

**Fig. 2:**
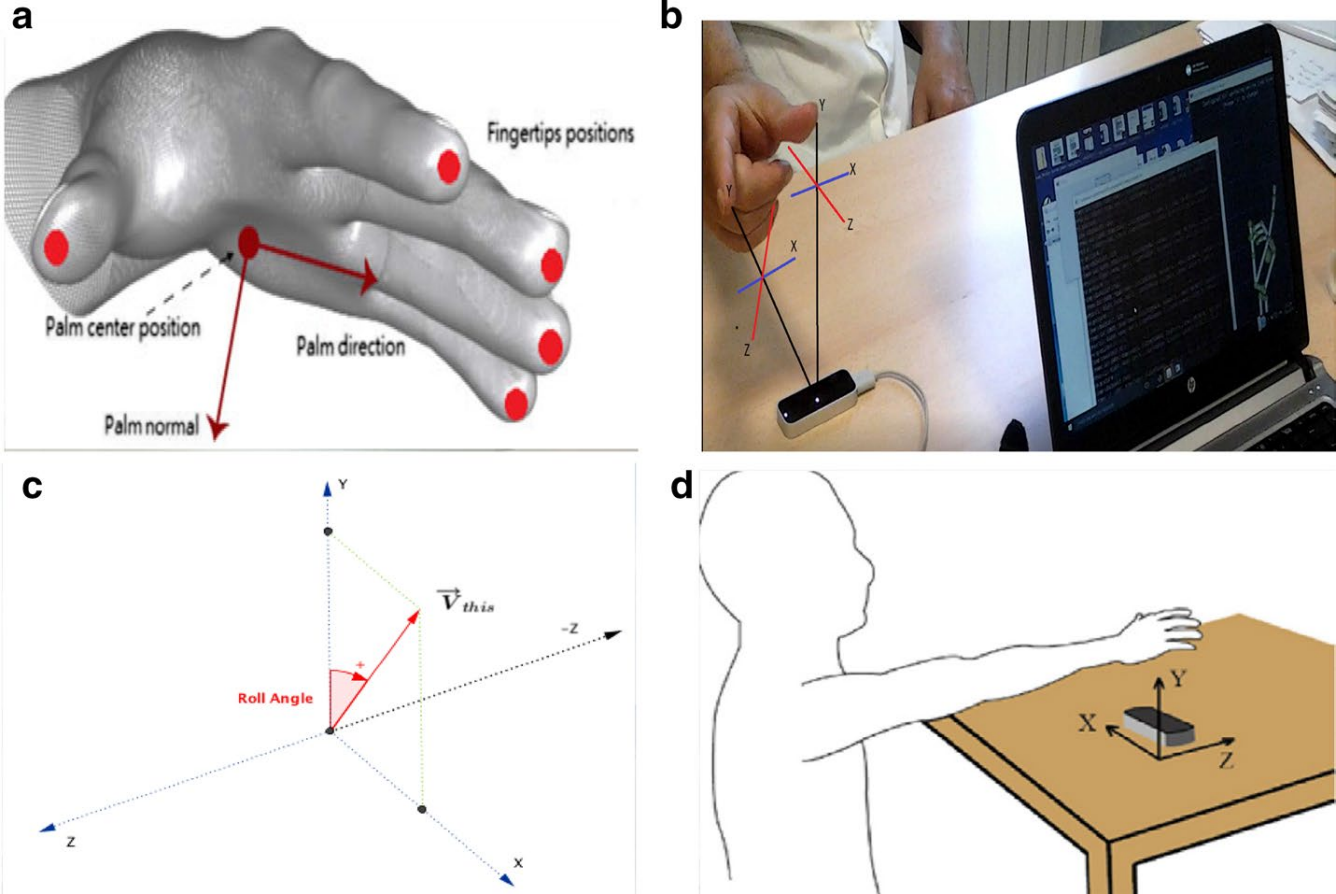
Leap Motion features on a gesture example: **a** fingertips distance [41], **b** thumb and index finger distance, **c** palm angle [21], **d** finger tips velocity [15]
2. Fingertip distance: Sum of each fingertip’s distance from the palm centre of hand.
  This value was used in the OPCL exercise and was computed by the LMC SDK instructions given below:
  2 $$D_{f} \left( k \right) = \mathop \sum \limits_{n = 1}^{5} fingers\left[ n \right].tipPosition.distanceTo\left( {hand.palmPosition\left( k \right)} \right)$$
  where n is the number of fingers.
  The hypothesis here is that the distance *D*_*f*_ is smaller in the closing position of the hands as compared to when all fingers are in the opening positions (Fig. 2a). Therefore, *D*_*f*_ provides adequate information about hand opening/closing movements.
3. Thumb forefinger distance: Distance between the thumb and index fingertips in pinch hand pose.
  This value was used in the THFF exercise and was computed via the LMC SDK instruction given below:
  3 $$D_{p} \left( k \right) = handPinchDistance\left( k \right)$$
  The distance is computed by looking at the shortest distance between the last two phalanges of the thumb and index finger. This pinch measurement only takes the thumb and index finger into account (Fig. 2b).
4. Fingertips velocity index: Average value of the velocity module of all five fingertips (Fig. 2d).
  This value was used in POST exercise and was computed via the LMC SDK instruction given below:
  4 $$V_{f} \left( k \right) = \frac{{\mathop \sum \nolimits_{n = 1}^{5} Finger_{n} .tipvelocity.y}}{5}$$
  This velocity index is demonstrated to be correlated to the tremor amplitude of the hand [17] and gives the rate of change of the fingertips and hand position.

Since the LMC does not have a constant frame rate, the collected data is non-uniform with respect to time. To satisfy the Shannon sampling theory [21] and to obtain consistency in feature extraction, all signals were reconstructed with a linear interpolation method with an average sampling rate of 50 Hz (Matlab 2015b).

### Feature extraction

The main features that are necessary to characterize the execution of the above listed exercises of the MDS-UPDRSIII included in this study are listed in Table 2.

**Table 2:** Biomechanical parameter extracted from all exercises

Exercises	Extracted features	Acronyms
PSUP	Number of rotational movements	num-PS
	Supination speed	wps
	Pronation speed	wsp
	Variability of frequency	fSD-PS
	Variability of amplitude	tetaSD-PS
OPCL	Number of opening/closing movements	num-OC
	Hand opening speed	wop
	Hand closing speed	wcl
	Variability of frequency	fSD-OC
	Variability of amplitude	tetaSD-OC
THFF	Number of thumb-forefinger tapping	tapTF
	Opening speed	woTF
	Closing speed	wcTF
	Variability of frequency	fSD-TF
	Variability of amplitude	tetaSD-TF
POST	Signal strength of the movement	PwrP
	Relative power in the band of interest of postural tremor (8–12 Hz)	PwrpP2

**Fig. 3:**
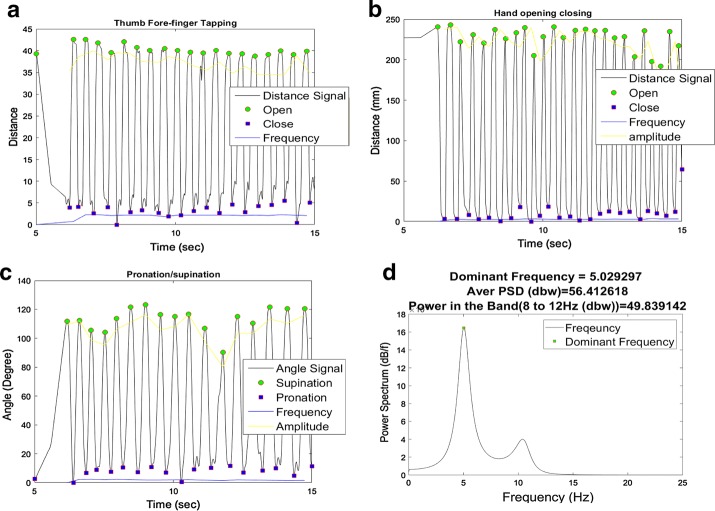
Example of the smoothing spline signal for all exercise as: **a** thumb fore-finger tapping, **b** hand opening closing, **c** pronation supination, **d** postural tremor. The frequency of each movement is obtained as the inverse of the time between consecutive peaks. The frequency of each movement was defined as the inverse of the time difference between the second and first peaks. The amplitude of each movement is obtained as the difference in amplitude from a peak to the next valley. Opening speed of hand and finger tapping were obtained with the distance travel between peak from the previous valley divided by time of hand or finger move from valley to peak. Similarly, for closing speed of hand or finger tapping were obtained with the distance travel between current peak to the next valley divided by time travel from current peak to the next valley. For pronation, angular velocity was obtained change in the angle from peak to last valley divided by time. Similarly, for supination, angular velocity was obtained with change in the angle from consecutive peak and valley divided by time duration of the change in angle from consecutive peak to valley. In postural tremor average velocity of fingers was used to obtain the signal strength and power in the band of interest 8–12 Hz. A Fast Fourier Transformation was used to obtain the power spectrum. Average of power spectrum was considered as the signal strength. Power b/w 8–12 HZ obtained with the average of power spectrum between 8 and 12 HZ. The tremor frequency is defined as the frequency with the maximum power in the spectrum

For PSUP, OPCL, and THFF, the number of movements included in the exercise and their velocity were calculated, as well as the variability of frequency and amplitude. To obtain the number of repetitions, an appropriate peak finder algorithm [22] was used to identify peaks and valleys in the signals based on the local maxima and minima. In PSUP and OPCL, peaks in *A*_*p*_ and *D*_*f*_ represent the respective supination and opening positions of hands, while valleys represent pronation and closing positions (Fig. 3a–c). Similarly, *D*_*p*_ was used for calculating the number of taps in THFF.

The amplitude of each single repetition is another important feature. It is calculated as the difference of the values of each peak and the next valley (Fig. 3) [23]. Furthermore, amplitude variability is estimated by calculating the standard deviations (std) of all individual movements of amplitudes.

Frequency of each movement is defined as the inverse of the time between consecutive peaks. Further frequency variability is also estimated by calculating the standard deviations of all individual frequency components, respectively.

Opening speed of hand or finger tapping in OPCL and THFF is defined as the distance variation between one peak from the previous valley divided by the time of hand or finger movement from the last valley to peak:

5 $$Speed_{open} = \frac{{Distance\, peak_{n} - Distance \,valley_{n - 1} }}{{Time \, peak_{n} - Time \, valley_{n - 1} }}$$

Similarly, closing speeds of hand and finger tapping are obtained with the distance variation between one peak to the next valley divided by the time of hand or finger movement from the last peak to the next valley:

6 $$Speed_{close} = \frac{{Distance \, peak_{n} - Distance \, valley_{n} }}{{Time \, peak_{n} - Time \, valley_{n} }}$$

Angular velocity in pronation is defined as the variation of the angle from one peak to the last valley divided by time. For pronation, angular velocity is defined as:

7 $$Angular \, Velocity_{pronation} = \frac{{Supination \,angle_{n - 1} - Pronation \, angle_{n} }}{{Time \, pronation_{n} - Time \, supination_{n - 1} }}$$

Angular velocity in supination is defined as the variation of the angle from one peak to the next valley divided by time duration.

For supination, angular velocity is defined as:

8 $$Angular \, Velocity_{supination} = \frac{{Supination \, angle_{n} - Pronation \, angle_{n} }}{{Time \, supination_{n} - Pronation \,angle_{n} }}$$

For the POST exercise, the $V_{f}$ signal was analyzed in the typical band of interest for Parkinson (8–12 Hz). Since the use of acceleration for tremor assessment is a more relevant variable than velocity or displacement [24], it was, first, time-differentiated to yield finger acceleration and then filtered with a low-pass Butterworth filter at 14 Hz [17]. Subsequently, the power spectral density (PSD) of the obtained signal acceleration was estimated using the Burg’s method in Matlab. Particularly, the following two features were estimated. The frequency with the maximum power was determined as the hand tremor frequency, and the average PSD in the band of interest (8–12 Hz) was obtained with the average of PSD of frequency components between 8 and 12 Hz. Results of the three trials were averaged to represent the performance of movement in the dominant hand (right) of the subject in all parameters.

### Feature analysis and selection

Feature selection was the next step to assess the potential of the extracted metrics and to select the most suitable parameters for machine learning classifiers to obtain the highest accuracy. From a statistical point of view, the Spearman’s correlation was estimated with SPSS21 (IBM, Armonk, North Castle, NY, USA) to determine the correlation between measured biomechanical parameters and clinical scores estimated by the neurologist. As proposed in [25], features with a correlation higher than strong (0.40–0.69: strong, 0.70–1.00: very strong) were considered as significant with respect to the clinical scores. Since the measurement for the PwPD group violated the normality assumption, the unpaired Mann–Whitney U test for non-parametric samples was calculated [26] to identify the ideal features for comparison between healthy controls and the PD group. The most dominant side (the side with the best task performance) of PD participants was compared with the participants’ best performing side of the healthy control group. The choice of the cut-off points to accept the alternative hypothesis (same as rejecting the null hypothesis) is totally subjective. The common use of p ≤ 0.05 was chosen by Fisher [27]. A probability value (p) of p < 0.05 was considered significant for all the analyses [28, 29]. ANOVA tests were used to assess differences in objective measurements between PwPD and control groups. Obtained results from ANOVA were presented in mean and standard deviation. All the statistical analyses were performed with SPSS21 (IBM, Armonk, North Castle, NY, USA). Simultaneously, Cohen’s d effect size was also calculated with an available online calculator [30].

Because the extracted features included considerable decreasing classification accuracy and the generalization of ‘‘noise’’ [4], we used the feature selection algorithms (WEKA 3.6, New Zealand) to select features with the most discriminative ability. In this study, we ran multiple tests using various algorithms and search methods (principal components analysis, support vector machine, consistency, J48, filtered subset evaluation, information gain, gain ratio, and Chi-square attribute evaluation) to define their optimal subsets and attributes, which would help us avoid over-fitting our model to redundant information (Table 5). All the methods were run over the tenfold cross validation. We used feature selection methods that were based on different criteria to define the value of single features and subsets of features. As is argued in [31], a single variable may not be important on its own, but it may contribute to the performance of the classifier when used in a subset [32].

### Classification algorithms for healthy control and PwPD

Supervised learning methods such as support vector machine (SVM), logistic regression (LR), and naive Bayes [32, 33] with tenfold cross validation were used to classify both groups of subjects (PwPD and control). The tenfold cross validation randomly splits the n different subjects into tenfolds with roughly proportional numbers of healthy and PwPD in each fold. The prediction algorithm is repeated 10 times with the cases of each fold withheld from the training set in turn, the cross-validated error rate being the average error rate on the withheld cases. A typical fold contained 10 subjects for the prostate data, healthy and PwPD, which were then predicted by the rule constructed from the data of the other remaining subjects. The SVM classifier was trained with sequential minimal optimization (SMO) methods and with polynomial kernel. All the classifiers were developed under the environment of machine learning software weka3.6 (University of Waikato, New Zealand).

## Results

### Correlation between objective parameters and subjective evaluation

The most significant parameters, characterized by a strong correlation with the clinical evaluations, are presented in Table 3. In all exercises the correlation between extracted parameters with the respective medical rank resulted mostly weak. On the same time, standard error of estimate showed that moderate values mean the moderate predication capability of extracted parameters. For the POST exercise, no correlation was found between the extracted features and the clinical rank.

**Table 3:** Spearman’s correlation between clinical scores and biomechanical parameters

Exercises	Extracted features	Spearman’s correlation	Standard error of estimate
PSUP	Num-PS	− 0.257	0.263
	Wps	− 0.009	0.254
	Wsp	− 0.025	0.211
	fSD-PS	− 0.488	0.199
	tetaSD-PS	0.307	0.257
OPCL	Num-OC	− 0.539	0.238
	Wop	− 0.647	0.281
	Wcl	− 0.639	0.264
	fSD-OC	0.313	0.244
	tetaSD-OC	− 0.647	0.280
THFF	tapTF	− 0.728	0.247
	WcTF	− 0.804	0.284
	WoTF	− 0.836	0.253
	fSD-TF	− 0.006	0.202
	tetaSD-TF	− 0.188	0.284
POST	PwrP	0.59	0.281
	PwrPR2	0.159	0.286

### Difference between the control and PwPD objective parameters

Assessment of the parameters (Table 4) between the PwPD and healthy control groups showed that Num-PS (number of pronation/supination), fSD-PS (frequency variation in pronation/supination), Num-OC (number of opening/closing hand), WOP (hand opening speed), WCL (hand closing speed), fSD-OC (hand opening/closing frequency variation), tetaSD-OC (hand opening/closing amplitude variation), Wc-TF (finger tapping closing speed), and Wo-TF (thumb forefinger tapping opening speed) showed a significant difference between the median of both groups of subjects (PwPD, control). At the same time, all the listed features have high effect size, so both groups (PwPD and healthy) have a large standard deviation and present significant differences. Other parameters—Wps (supination speed), Wsp (pronation speed), and tetaSD-PS (frequency variation in pronation/supination)—showed statistical significance according to Mann–Whitney but had small effect size, which means that both groups (healthy and patients) are not differ from large standard deviation. Similarly, from postural tremor, PwrP (signal strength) and PwrpP2 (signal strength between 8 and 12 Hz) parameters were not significant according to Mann–Whitney and Spearman correlation, and they also presented a small Cohen’s effect size.

**Table 4:** Mann–Whitney statistical significance between patients and healthy controls

Exercises	Extracted features	Control	Patient	p	Cohen’s d effect size
PSUP	Num-PS	18.82 ± 5.52	15.08 ± 4.283	0.034	0.757
	Wps	173.71 ± 47.91	181.35 ± 74.9	0.509	− 0.1215
	Wsp	179.97 ± 47.162	187.45 ± 77.2	0.509	− 0.1164
	fSD-PS	0.79 ± 0.225	0.54 ± 0.132	0.001	1.3553
	tetaSD-PS	16.92 ± 5.709	15.29 ± 11.96	0.044	0.173
OPCL	Num-OC	19.83 ± 3.102	16.50 ± 5.38	0.002	0.758
	Wop	378.78 ± 71.87	322.54 ± 125.9	0.059	0.5486
	Wcl	378 ± 71.87	322.54 ± 125.9	0.059	0.5486
	fSD-OC	18.75 ± 6.42	14.75 ± 8.58	0.059	0.5278
	tetaSD-OC	0.6242 ± 0.129	0.530 ± 0.123	0.065	0.747
THFF	tapTF	22.58 ± 6.798	23.55 ± 10.31	0.300	− 0.111
	WcTF	87.29 ± 43.515	116.39 ± 56.5	0.073	− 0.5770
	WoTF	85.64 ± 39.524	111.70 ± 50.5	0.087	− 0.5747
	fSD-TF	10.26 ± 6.320	8.89 ± 4.233	0.284	0.2547
	tetaSD-TF	0.87 ± 0.239	0.86 ± 0.269	0.379	0.0399
POST	PwrP	72.68 ± 16.618	91.17 ± 54.29	0.161	− 0.4605
	PwrPR2	55.748 ± 6.583	63.950 ± 28.80	0.274	− 0.3926

### Feature selection for a machine learning model

Before training the machine learning classifier, a number of multiple tests, based on various algorithms and search methods, were used to select the most significant features. This preliminary study was crucial to define optimal subsets and attributes, which could help avoid over-fitting the machine learning model with redundant information, and avoid a high-dimensional features space, which may affect the ability of the machine learning system to successfully classify.

The reason for using the multiple feature selection methods was because a single variable may not be important on its own, but its contribution to the performance of the classifier could be relevant when used in a subset. In Table 5, the feature selection approaches, based on subset space (principal components analysis, support vector machine, information gain, gain ratio, and Chi-square attribute evaluation), frequently have similar results, in terms of selected features, to the selection approaches, based on attribute space (consistency, J48, and filtered subset evaluation), even if there are noticeable differences between the two methodologies. For example, the Num-PS feature was selected by the two approaches, subset space and attribute space methods, but it was ranked differently (i.e. lower in the first and higher in the second). On the other hand, the PwrP feature was considered more important by most of the attribute space-based methods and not relevant by the other space-based subsets. Regardless of the method used, the Num-OC feature was almost always deemed important during the feature selection tests, suggesting that it was useful as a discriminating feature.

**Table 5:** Features selection test according to feature selection methods

Test number	Method	Selects	Search algorithms	Selected subsets/features (mean value among the three trials)
1	Principal components	Attributes	Ranker	Num-OC, Wcl, tetaSD-OC, Wop, Num-PS
2	SVM	Attributes	Ranker	fSD_PS, WcTF, tetaSD-PS, tetaSD-OC, Wps, WoTF, Num-PS
3	Consistency	Subset	Greedy SW	Num-OC, Wop, fSD-PS, tetaSD-PS
4	J48	Subset	Greedy SW	Num-OC, WcTF, Wop
5	Filtered subset evaluation	Subset	Genetic search	Num-PS, Num-OC, tetaSD-PS, fSD-PS, Wcl
6	Information gain	Attributes	Ranker	PwrP, fSD-TF, Num-OC, tetaSD-TF, Wsp, Wps, Num-PS
7	Gain ratio	Attributes	Ranker	PwrP, fSD-TF, Num-OC, tetaSD-TF, Wsp, Wps, Num-PS
8	Chi square attribute evaluation	Attributes	Ranker	PwrP, fSD-TF, tetaSD-TF, Num-OC, Wsp, Wps, Num-PS

Out of 17 derived features, 14 were considered appropriate for the classification models. Three features, i.e. fSD-OC, tapTF, and PwrPR2, were not selected by any feature selection methods, thus appearing trivial for the classification between the PwPD vs. healthy subjects.

### Classification performance

Three different machine learning classifiers were used, namely logistic regression (LR), naïve Bayes (NB), and support vector machine (SVM), to establish the best performing method. For each subset of features, selected in Table 5 (numbered from 1 to 8), the three classifiers were applied with the tenfold cross validation method. The performance of each subset with respect to each classifier (LR, NB, SVM) is presented in Tables 6, 7 and 8, respectively.

**Table 6:** Logistic REGRESSION CLASSIFICATION test

Classifier	Average accuracy (%)	AUC	TP	TN	Test number
LR	44.44	0.339	25	60.0	1
	70.37	0.831	58.3	80.0	2
	62.93	0.672	50.0	73.3	3
	66.66	0.65	50.0	80.0	4
	59.25	0.578	41.0	78.0	5
	55.5	0.572	41.7	66.7	6
	55.55	0.572	50.0	60.0	7
	55.55	0.572	41.7	66.7	8

**Table 7:** Support vector machine classification test

Classifier	Average accuracy (%)	AUC	TP (%)	TN	Test number
SVM	40.74	0.40	53.00	66.7	1
	70.37	0.675	41.7	93.3	2
	66.66	0.642	41.7	86.7	3
	59.25	0.558	25.0	86.7	4
	74.07	0.717	50.0	93.3	5
	51.85	0.492	25.00	73.3	6
	55.85	0.525	25.00	80.0	7
	51.85	0.492	25.00	73.3	8

**Table 8:** Naïve Bayes classification test

Classifier	Average accuracy (%)	AUC	TP	TN	Test number
NB	51.8	0.589	58.3	46.7	1
	81.4	0.811	75.0	86.7	2
	74.0	0.8	75.0	73.3	3
	62.9	0.171	75.0	53.0	4
	74.4	0.783	75.0	73.3	5
	55.5	0.533	75.0	40.0	6
	48.1	0.539	66.7	33.3	7
	55.5	0.533	75.0	60.0	8

The best performing classifier was the NB, achieving 81.45% of average accuracy, 76% of sensitivity, 86.5% of specificity, and a 0.811 AUC value, using a selection of features from the feature subset defined by a SVM ranker method (number 2 in Table 5). The same feature subset performed slightly worse with the other classifiers (SVM and LR), with an accuracy, sensitivity, and specificity of 70.37%, 41.7% and 93.3%, respectively, for SVM and of 70.37%, 58.3% and 80.0%, respectively, for LR. All the other subsets, selected by the other feature selection methods, showed the worst classification performance in all machine learning classifiers (LR, NB, SVM), as shown in Tables 6, 7 and 8, respectively.

## Discussion

This study investigated the possibility to use the LMC as an assessment tool in diagnosis and monitoring of Parkinson disease. Particularly, the four main exercises of the UPDRS Part III clinical protocol involving only upper limbs (PSUP, OPCL, THFF, and POST) were used to test the LMC device, and a comparison with the relative clinical score provided by the neurologist was performed, as well as a comparison with a healthy control group. To the best of our knowledge, this is the first work that tried to study the feasibility of a low-cost 3D camera-based marker less hand tracking system in clinical settings with patients with Parkinson disease and with a clinical standardized protocol.

In the PSUP exercise, the number of rotation movements (Num-PS), frequency (fPD-SD), and amplitude (tetaSD-PS) variability showed good significative for the distinction of patients and healthy control in terms of p-test and Cohen’s d coefficient (Table 4). Interestingly, these parameters were also included, as selected features, in the most performant selection feature method, the SVM ranker method (Table 5), used in combination with the NB machine learning model.

Supination speed (Wps) during the pronation/supination contributed significantly, as it was selected frequently in different feature selection methods. Similarly, in PwPD, the speed should be discontinuous due to fatigue, freezing, and uncontrollable rotational movements, should eventually reduce the supination speed, and should contribute significantly toward classifying the healthy and control subjects as frequently selected by feature selection methods. Conversely, no clinical association was revealed, which directs the intra-rater variability of the clinical scores. Low effect size also directs the limited contribution of the parameter to classify the PwPD and healthy control.

Statistically significant differences or outcomes simply address whether to accept or reject a null or directional hypothesis, without providing information on the magnitude or direction of the difference (between groups). To improve the interpretation of clinical significance, researchers commonly include more clinically-relevant information such as confidence intervals and effect sizes, which reflects the magnitude of the difference in outcomes between groups. A greater effect size indicates a larger difference between PwPD and control groups. Statistically significant differences alone should not be the primary influence for clinical interpretation of a study’s outcome for application to patient care. Hence, it proves that even if some features show statistical significance, it is not necessary it also reflect the clinical significance, which is often misinterpreted. The additional analysis to investigate the clinical significance is mandatory via methods such as effect size [34].

Amplitude variation (tetaSD-PS) in the pronation/supination, selected by two feature selection methods (Table 5: 2, 3, 5), showed significance. This parameter also revealed a clinical association in the previous study [35]. The roll angle metrics showed the potential to measure the metrics which can extract characteristics related to bradykinesia and hypokinesia and thus can discriminate between healthy individuals and PwPD. Standard deviation was used to determine the strength of the signal, so the higher values of STD indicate stronger movements [35]. LMC showed potential to quantify the amplitude variation in the forearm-based rotational movements, which also contributed to classifying the healthy and PwPD subjects.

In the hand opening/closing, four parameters—Num-OC (number of hand opening/closing movements), Wop (hand opening speed), Wcl (hand closing speed), and tetaSD-OC (hand opening/closing amplitude variation)—showed significant clinical association while also showing a high effect size between two groups (patients and healthy subjects). In feature selection methods, these parameters were selected frequently, demonstrating that these features have potential to contribute significantly to classifying the PwPD and healthy subjects. The average distance of all fingertips from palm positions was exploited to extract the other related parameters. This study revealed other potential metrics such as Wop (hand opening speed), Wcl (hand closing speed), and tetaSD-OC (hand opening/closing amplitude variation), which were not consistent with a previous preliminary study on LMC [35].

Hand opening/closing amplitude variation (tetaSD-OC) not only was selected by feature selection methods, but it also showed clinical association with the neurologist’s assessment. Rhythm is a characteristic mentioned in the MDS-UPDRS and can be defined as any sequence of regularly reoccurring events. The STD of estimated amplitude of the movements represented this characteristic and contributed significantly to classify the PwPD and healthy subjects, which is also consistent with the pervious preliminary studies on LMC [35].

In addition, in the thumb forefinger tapping (THFF), most of the features showed good performance in the clinical correlation and distinction between the two groups. Bradykinesia refers to movement that is slower than desired. These symptoms can be assessed with repetitive movements. Repetitive movements in the finger tapping motion result in a progressive reduction in tapping speed and motion amplitude, and they increase the use of visual feedback as a compensatory mechanism for a motor system with inherently high variability of motor output [36]. Particularly, WoTF and WcTF were selected in the most performant feature selection methods and also presented a good clinical association for clinical assessment, thus being potentially able to discriminate the bradykinesia. Feature selection methods also ranked highly. It is known that repetitive finger tapping movement in bradykinesia/hypokinesia eventually reduces in speed due to fatigue, and thus the movement is helpful in discriminating between healthy individuals and PwPD.

In the OPCL and THFF, most of the parameters demonstrated both a good correlation with the clinical assessment (Table 3) and good significance in distinguishing between patients and healthy subjects (Table 4). Conversely, the same cannot be said of the PSUP and POST exercises, showing that in this case, there could be some limitations in the capability of the LMC to be used as a clinical assessment tool. This latter point was preliminarily found in another study [33] and also better investigated in [37], which revealed that the LMC could not return clinically meaningful data for measuring forearm pronation/supination, having serious inconsistencies in reported joint angles (RMSE = 38.4°). Therefore, the functional SDK measurement accuracy of the frequency, amplitude, and speed of such movement, described by the angle between the negative y-axis and the projection of the vector on to the x–y plane (roll-angle), was not sufficient and did not match with the observation and estimation of the clinician. Additionally, this low accuracy could be further worsened by the fact that the PSUP exercise was performed by patients in an advanced stage of the disease who had difficulty in performing regular movements of pronation and supination at fastest velocity, thus contributing to increasing the error in measuring the PSUP parameters [38]. However, it should be considered that, despite some accuracy problems related to the roll angle metrics, the other parameters appropriately contributed to the general assessment, and this was also confirmed by the results of the feature selection methods (Table 5). These other parameters are those that are not connected with the necessity to measure accurate angles and, mainly, concern those value related to counting something, i.e. number of taps, rotations, etc. Indeed, these parameters do not require accuracy in the angles, but only track the variation of the signals that enables counting the execution of the movement, i.e. in pronation/supination peak-to-peak movements (Num-PS) was a parameter that was not influenced by the accuracy of the angle estimation. This lack of accuracy in measuring some parameters may lead to a similar situation in tremor exercise (POST). Here, the parameters were calculated from the velocity signal, which in the posture exercise referred to a very small movement, thus requiring high accuracy.

The thumb forefinger tapping also revealed potential metrics such as tapTF (thumb forefinger tapping opening/closing movements), which is not consistent with a preliminary study on LMC [35]. In contrast, small effect size and exclusion of tapTF from the feature selection method directs the biased clinical association of this parameter. One significant reason could be intra-rater variability in the clinical scores. Two other parameters (Wotf, Wctf) also showed a clinical association, while a large effect size endorse the significance of these parameters. The fact that feature selection methods also selected these features frequently endorses the significance of these parameters. Frequency and amplitude variation in the finger tapping did not show clinical association—in addition, low effect size in both parameters between two groups (PwPD and healthy control) also showed the insignificance of these parameters. In postural tremor, no parameter showed clinical association, while small effect size also showed the limited potential of these parameters to provide clinically meaningful information.

## Limitations

One of our main goal in this study was to investigate matrices that could be extracted from a MDS-UPDRSIII tasks, and to confirm its merit when combined with the other well-established metrics included in our analysis. Because these metrics could lead to highly correlated features, it is not appropriate for all of them to be used in the same classification model. Therefore, during our treatment of the data with machine learning techniques, we opted against the complexity of re-introducing feature selection inside the classification training, and assessed the performance of the individual feature subsets, as they are given in Table 5, applying various classifiers after the feature selection procedure, as shown in Tables 6, 7 and 8.

Maximizing the generalize ability of machine learning in neuroscience will require a different type of validation approaches. In other words, do the machine learning discovered models capture fundamental principles of brain function and reflect causative phenomenon that extrapolate across multiple biologically relevant contexts [39]. On the same time testing the different and new data on the same training model is next step to obtain the highest accuracy for the diagnosis. Since, supervised machine learning techniques have been extensively used in predicting PD through a set of datasets. However, the most methods developed by supervised methods do not support the incremental updates of data [40]. Even we test the new data on final training set, whenever there is fresh data there is need to update the training model. Moreover, feature selection every time for a new training model does not allow to automate and implement this model for real time applications. Ultimately there is need to investigate machine learning techniques which supports incremental updates and re-learning of data. In recent years, convolutional neural networks (CNNs) have shown excellent performance on classification problems when large-scale labeled datasets are available. However, it is challenging to apply CNNs to problems where only small labelled datasets are available. For example, collecting and labeling a large amount of medical data is often difficult. As a result, it is challenging to apply CNNs to small-scale medical data. To enable automated evaluation of PD motor states which covers a wide range of PD symptoms across patients, a large amount of wearable sensor data in daily-living conditions is needed [41].

## Conclusions

In this study, highly reliable metrics provided by the Leap Motion SDK for skeletal tracking were exploited for MDS-UPDRSIII motor tasks to assess bradykinesia-related characteristics. This study, for the first time, focused on the MDS-UPDRSIII motor tasks. Other authors [42, 43] investigated the potential of the LMC for rehabilitation of the palm and fingers for patients with cerebral strokes, physical injury, or other developmental disabilities. Others [37, 44–46] focused on investigating the clinical association of the extracted information. Our approach required both PwPD and healthy subjects to perform MDS-UPDRSIII motor tasks from in a clinical environment. At the same time, one neurologist visualized the performance of each subject and assigned a subjective score based on the performance of the subject. The LMC-provided SDK was exploited to extract the related parameters for objective assessment of motor dysfunction in PwPD. Our finding showed that LMC has a limited angle of view to assess the motor dysfunction in PwPD. Certain gestures could not be recorded properly depending on the placement of the Leap Motion controller [46]. The sensor was not able to recognise all of the fingers. Fingers touching each other, folded over the hand, or hidden from the camera viewpoint were not captured, and in many configurations, some visible fingers could be lost, specifically if the hand is perpendicular to the camera [47], eventually effecting the accuracy of the measured information. Experimental results showed that although the data recorded from Leap Motion was not completely reliable, a reasonable overall accuracy with the proposed set of features and classification algorithms was obtained. In general, we can conclude that LMC is not yet able to track motor dysfunction characteristics from all MDS-UPDRS proposed exercises. However, intra-rater variability in clinical scores could be one significant reason for the limited monotonic relationship between clinical scores and biomechanical parameters when classifying with machine learning algorithms. It would be interesting to investigate the other potential metrics to extract the related parameters. Following the same methodology with a large number of samples and more than one evaluator (neurologist) and employing only the tasks where consensus is found, such as hand opening/closing, could lead to an unbiased objective measuring system. This could lead to an improvement in the assessment and monitoring of movement disorders using LMC. This study, for the first time, revealed the functional limitation of LMC-provided SDK for postural tremor assessment in PwPD. One possible reason could be an inconsistent frame rate of the device with respect to time. Moreover, it is important to enhance the accuracy in SDK algorithms. Usability of the workspace should be improved to facilitate the use by nontechnical individuals.

Finally, it is possible to state that the LMC represents a very promising device for the implementation of innovative service [48] in neurodegenerative diseases and other ageing-related disorders or frailties; however, improvements in SDK algorithm accuracy and usability are required for consolidating its use in healthcare contexts.
